# Correction: ‘Redox-mediated carbon monoxide release from a manganese carbonyl—implications for physiological CO delivery by CO releasing moieties’ (2021), by Barrett *et al.*

**DOI:** 10.1098/rsos.231206

**Published:** 2023-09-11

**Authors:** Jacob A. Barrett, Zhi Li, John V. Garcia, Emily Wein, Dongyun Zheng, Camden Hunt, Loc Ngo, Lior Sepunaru, Alexei V. Iretskii, Peter C. Ford

**Keywords:** hydrogen peroxide, manganese carbonyl, CO releasing moiety, redox reaction

*R. Soc. Open Sci.* **8**, 211022. (Published online 10 November 2021). (https://doi.org/10.1098/rsos.211022)

Error in the sentence:

‘If one assumes cellular volumes of approximately 2 pl [63], this translates into approximately 6 moles l^−1^ h^−1^.’

The correct sentence should read:

‘If one assumes cellular volumes of approximately 2 pl [63], this translates into approximately 25 mmoles l^−1^ h^−1^.’

The original statement inaccurately reported the conversion from cellular volumes to molar concentration. The correct value is 25 mmoles l^−1^ h^−1^, not 6 moles l^−1^ h^−1^ as mentioned in the published version.

This has been corrected on the publisher's website.

